# Supplementary figures and images for: Emergence of Two Distinct SARS-CoV-2 Gamma Variants and the Rapid Spread of P.1-like-II SARS-CoV-2 during the Second Wave of COVID-19 in Santa Catarina, Southern Brazil
Source: Viruses. 2022 Mar 27;14(4):695. doi: 10.3390/v14040695 (PMC9029728; doi:10.3390/v14040695)

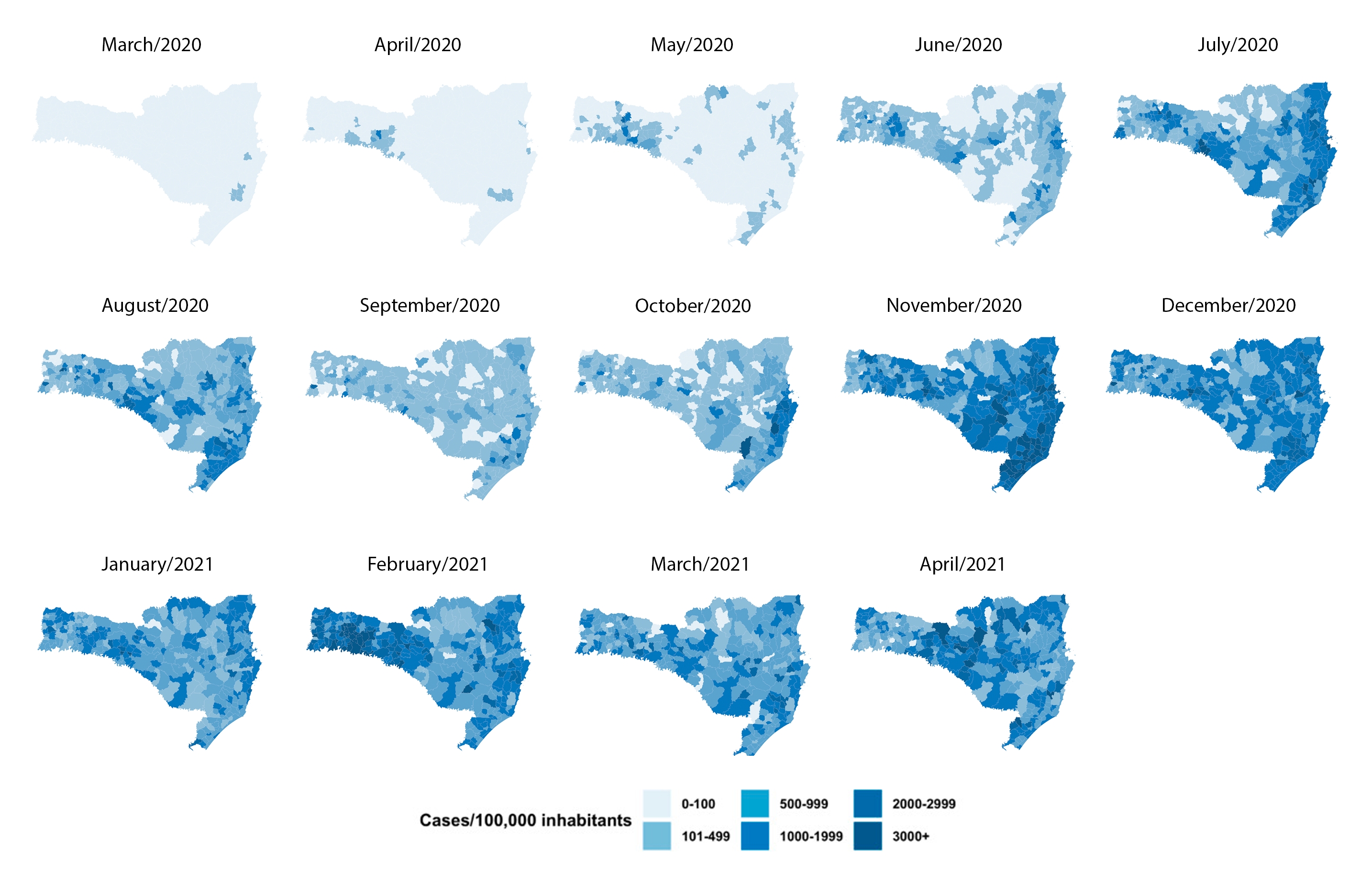

Supplement: Supplementary file 1 [file viruses-14-00695-s001.zip › viruses-1634576-supplementary/Supplementary_Fig_Table/Figures/Supplementary Figure 1.jpg]

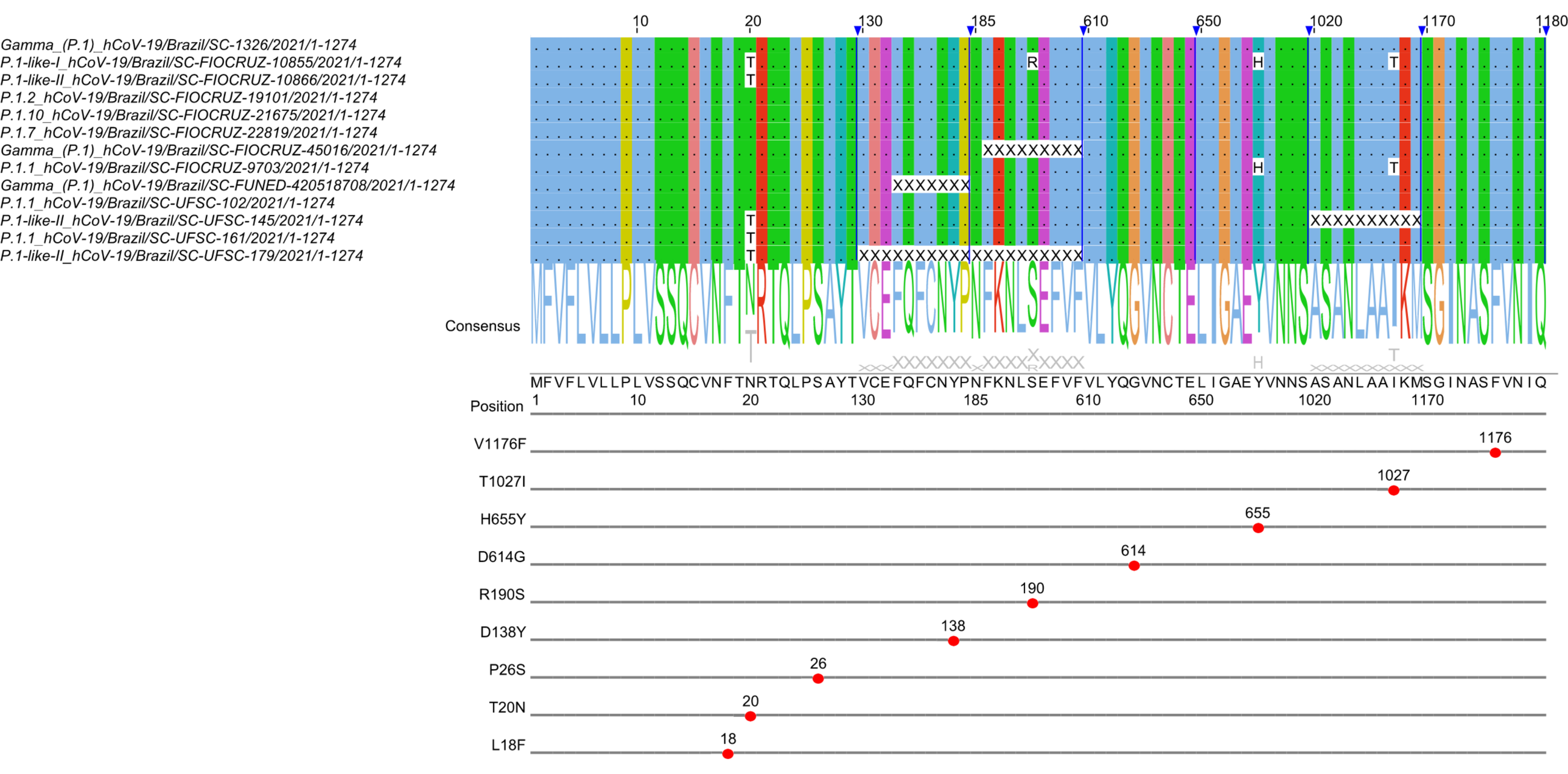

Supplement: Supplementary file 1 [file viruses-14-00695-s001.zip › viruses-1634576-supplementary/Supplementary_Fig_Table/Figures/Supplementary Figure 2.jpg]
